# Clinically-driven design of novel methods of investigation on skeletal health status in neurological disorders. The case of the traumatic brain injuries

**DOI:** 10.3389/fneur.2023.1176420

**Published:** 2023-05-17

**Authors:** Letizia Penolazzi, Sofia Straudi, Nicola Lamberti, Elisabetta Lambertini, Chiara Bianchini, Fabio Manfredini, Roberta Piva

**Affiliations:** Department of Neuroscience and Rehabilitation, University of Ferrara, Ferrara, Italy

**Keywords:** traumatic brain injury, skeletal system, 3D cell culture, ectopic ossification, osteoporosis

## Introduction

Neurological disorders come in many forms which include infections, trauma, stroke, seizures, tumors, autoimmune and neurodegenerative conditions. All these diseases have temporary or permanent impacts on different districts of the entire organism. Most of the research in the field of brain diseases has focused on neurocognitive and neuropathological changes, while little is known about the long-term consequences on peripheral tissues including skeletal system.

The bidirectional connections between brain and bone or brain and joint have been widely described in terms of soluble factors and molecular mechanisms that participate both in the maintenance of physiological homeostasis and in the onset of various diseases affecting these tissues (1–4). It is possible to try to answer the many open questions in this field through the development of new basic research methods rigorously guided by clinical approaches.

Here we would like to express our opinion on the importance of designing adequate experimental models to understand the effects that traumatic brain injury (TBI) may have on skeletal system whose state of health is fundamental for the recovery of ambulation and mobility. TBI occurs as a result of an external force, typically caused by falls, accidents, sport activities, and military conflicts (5, 6). TBI with varying degrees of severity affects annually millions of individuals worldwide, across all ages (7). TBI patients continue to suffer for a long time from neurological and physical impairments that have a major catabolic effect on different parts of the body, known as polytrauma (8, 9).

## Traumatic brain injury and bone health

After TBI, bone health is impaired with rapid imbalance between bone formation and resorption which is supported by inflammation, endocrinologic stress-related response and pharmacologic side effects (10, 11). Moreover, an abnormal bone formation, the so called neurological heterotopic ossification, may affect the soft tissues around articular joints inducing pain, nerve compression, and movement restrictions (12, 13).

Unfortunately, post-TBI bone metabolism abnormalities may not be detected until considerable skeletal damage has already occurred, making neuro-orthopedic interventions to restore flexibility and range of motion more difficult. It is critical to identify targeted strategies that can reduce the rate of TBI-mediated secondary conditions to improve the individual's quality of life and lower costs associated with long-term health care and disability (6, 13).

It is known that bone homeostasis and remodeling are under the control of neuroendocrine and neuronal signals originating from the brain (3, 14, 15). Therefore, it is not surprising that this control is compromised following the hypothalamus–pituitary axis is disrupted, and blood brain barrier integrity is lost (16, 17). Despite numerous preclinical and clinical studies, the evidence and mechanistic data supporting bone and cartilage changes after TBI remain unclear and, in some cases, difficult to interpret (13, 18). Post-TBI, both osteopenia and osteoporosis may occur due to decreased bone mineral density (11, 19–21), but also accelerated fracture healing and enhanced callus formation, or even ectopic ossification (18, 22, 23). These opposing conditions can be explained by various factors and phenomena which, to a different extent for each patient, can cause long-term consequences of TBI on remote organs such as bone. Among these it is worth mentioning: extent of brain damage, age, gender, humoral factors, neuropeptides, molecules triggering specific signaling pathways (e.g., IGF-1, IL-6, IL-11, IL-18, GH, PTH, Wnt), cells (e.g., resident immune cells, neurons, osteoblasts, osteoclasts), stress-mediated activation of hypothalamus–pituitary–adrenal axis, lifestyle (e.g., falls, malnutrition, physical inactivity, vitamin D deficiency) (1). Unfortunately, due to the heterogeneity of the brain injuries, most of the current clinical studies are only partially informative on the impact of the numerous parameters mentioned, and furthermore it must also be emphasized that it is complicated to recapitulate the human condition in preclinical TBI animal models. In any case, the evidence collected so far has begun to shed light on the patient-dependent activation of those injury cascades (neuroendocrine humoral outflow and inflammatory mediator release) that may mediate secondary effects at bone level, partly explaining the broad spectrum of post-TBI skeletal deterioration reported in the literature (1).

## A 3D “bone-like” experimental model for TBI patients

To develop a more comprehensive clinical treatment including improvement of bone health in TBI patients, it will be necessary to strengthen the concept of patient-oriented therapeutic strategies. With this perspective it must be taken into account that animal models help us to understand the key mechanisms of TBI-induced bone alterations only partially, as they have a different anatomy and posture from the human one and consequently are not exactly appropriate for obtaining informative data on pathophysiological aspects of human bone and joint tissue (24, 25). In our opinion it is useful to try to develop *ex vivo* culture models based on TBI patient bone cells which, suitably combined, mimic the bone microenvironment as closely as possible. Although a “bone-like” complete model has not yet been produced, the evidence from several examples of three dimensional (3D) *in vitro* bone models is encouraging (26, 27) both for disease modeling and drug screening. Regarding TBI, this perspective may be relevant to obtain new evidence both to better understand the characteristics of bone cells of TBI patients, and to develop reparative/regenerative therapies aimed at preventing or slowing down adverse skeletal changes. Here we propose to evaluate the possibility of setting up a 3D “bone-like” model with the patient's own cells to create an *in vitro* bone microenvironment to cover methodological gap mentioned above. The TBI patients, depending on the severity of the damage and the affected areas, can undergo various surgical procedures involving the axial skeleton (bones of the skull, ossicles of the middle ear, nose, hyoid bone of the throat, vertebral column, and the thoracic cage) and the appendicular skeleton. Therefore, the surgical fragments obtainable from each of these districts (some of which—ear or nose—particularly easily accessible) can be good sources for isolating bone-forming cells, osteoblasts, and cartilage-forming cells, chondrocytes. The cell population resulting from these fragments may also contain progenitor cells at different stages of maturation and mesenchymal stem cells, optimal therapeutic targets for functional recovery. As previously demonstrated, this cell population may be co-cultured with the monocytes which are the progenitors of bone-resorbing cells, the osteoclasts, easily obtainable from a small amount of peripheral blood (28–30). When grown in a scaffold-free culture medium, osteoblasts and osteoclasts tend to form a 3D aggregate which remains viable for at least 14 days, the sufficient time to conduct the necessary experiments. Obviously, this co-culture system does not achieve the complexity of the bone microenvironment and many aspects need to be improved, however it represents a valuable tool to better understand the biology of bone cells for developing tailored therapeutics (31–33). Since the culture period is short, it is reasonable that the cells feel the conditioning of the source from which they were obtained in terms of epigenomic profile and secretome, generating a patient-specific response to certain stimuli/treatments (34–36). This issue is involving several scientists who are studying how soluble and insoluble extracellular cues are integrated and stored during the life of a cell. Among the large variety of protein and lipid factors released by the brain after TBI are those that have a significant impact on bone tissue (37–39) and may therefore continue to affect primary bone cells in culture. Another aspect that should not be underestimated is the role of genetic factors that could drive the interindividual variability of outcome following TBI (40, 41) and consequently the behavior of bone cells *in vitro*. Therefore, bone cells from a TBI patient can generate a “diseased” co-culture system with different behaviors not only compared to those of a “normal” system generated by healthy donor cells (obtainable following surgery for a fracture), but also compared to that of other TBI and non-TBI patients. The behavior of the cells in the 3D co-culture after exposure to a specific chemical or physical treatment (different oxygen percentage or mechanical stimuli) can be evaluated monitoring the secretome, the expression of specific genes, and specific functionalities such as the ability to differentiate, resistance to apoptosis, and cell-cell and cell-matrix interactions. This is certainly a challenge considering the low number of cells that can be obtained from biopsy of TBI patients. However, the advancement of technology allows the co-culture system to be translated to more sophisticated and miniaturized culture conditions with the employment of organ-on-a chip and integrated microfluidic culture platforms or bioreactors for dynamic culture conditions (27, 28, 42–46). Interestingly, the co-culture system we proposed can be subjected to external mechanical stimulation (tension, shear stress, compression, and hydrostatic pressure) similar to that experienced by the human skeleton by using various methods, including acoustic waves and magnetic field (47, 48). The responsiveness of the patient's cells to these stimuli can be evaluated at molecular level giving an indication of the probability of success of a specific rehabilitation treatment or physical exercise. It is known that physical activity and exercise can improve bone healing by inducing favorable vascular, hormonal and neural adaptations through mechanical, physical and biochemical perturbations. Therefore, it is interesting to be able to combine *in vitro* cellular and molecular observations with the clinical study of the effects on bone status of progressive loading phases, structured microdoses of exercise or periods of physical activity, depending on the severe, moderate or mild stage of TBI. Indeed, this could offer new perspectives in the study of bone reparative or remodeling processes mediated by rehabilitation and in the development of personalized mobilization loads or well-dosed exercise.

## Discussion

With this contribution we wanted to express our opinion on the need to develop new experimental *in vitro* models to try to shed light on apparently contradictory aspects concerning the effects that TBI can have on skeletal health. We think that the 3D co-culture system based on patient's bone cells may represent an opportunity not only to better understand the bone biology, but also to test the activity of catabolic or anabolic drugs on osteoblasts or osteoclasts in a personalized manner taking into account of the pathophysiological characteristics of the patient's cells. Therefore, such an osteoblasts/osteoclasts platform can help to develop patient-tailored therapeutic strategies aimed at reducing the osteoporosis or the ectopic ossification in different TBI patients. Importantly, this approach can also provide useful insights into fracture healing process and various diseases affecting bone and joint in non-TBI patients, reducing costs and time consumption.

It is clear that each experimental model has advantages and limitations and, as far as bone and cartilage tissue are concerned, there are interesting reviews in the literature describing the pros and cons about of different experimental model systems (26, 44, 49, 50). We believe that the clinically-driven design of both *ex vivo* culture systems, as the “bone-like” experimental model here described, and *in vivo* animal models allow exploiting the unique advantages of each leading to the best outcomes for treating skeletal complications even in TBI patients.

## Author contributions

RP, LP, and FM conceived the idea. RP and FM prepared the text and generated the final form with active support from SS, NL, EL, and CB. All authors contributed to the article and approved the submitted version.
